# Twitter-Based Social Support Added to Fitbit Self-Monitoring for Decreasing Sedentary Behavior: Protocol for a Randomized Controlled Pilot Trial With Female Patients From a Women’s Heart Clinic

**DOI:** 10.2196/20926

**Published:** 2020-12-04

**Authors:** Marily Oppezzo, Jennifer Tremmel, Manisha Desai, Michael Baiocchi, Danielle Ramo, Mark Cullen, Judith J Prochaska

**Affiliations:** 1 Stanford Prevention Research Center Stanford University School of Medicine Stanford, CA United States; 2 Interventional Cardiology Women's Heart Health at Stanford Stanford, CA United States; 3 Quantitative Science Unit Stanford University School of Medicine Stanford, CA United States; 4 Epidemiology and Population Health Stanford University School of Medicine Stanford, CA United States; 5 Department of Psychiatry and Weill Institute for Neurosciences University of California San Francisco San Francisco, CA United States; 6 Hope Lab San Francisco, CA United States; 7 Primary Care and Population Health Stanford University School of Medicine Stanford, CA United States

**Keywords:** support group, sedentary behavior, eHealth, Twitter, Fitbit, intervention, behavior change theory, mobile phone

## Abstract

**Background:**

Prolonged sitting is an independent risk behavior for the development of chronic disease. With most interventions focusing on physical activity and exercise, there is a separate need for investigation into innovative and accessible interventions to decrease sedentary behavior throughout the day. Twitter is a social media platform with application for health communications and fostering of social support for health behavior change.

**Objective:**

This pilot study aims to test the feasibility, acceptability, and preliminary efficacy of delivering daily behavior change strategies within private Twitter groups to foster peer-to-peer support and decrease sedentary behavior throughout the day in women. The Twitter group was combined with a Fitbit for self-monitoring activity and compared to a Fitbit-only control group.

**Methods:**

In a 2-group design, participants were randomized to a Twitter + Fitbit treatment group or a Fitbit-only control group. Participants were recruited via the Stanford Research Repository System, screened for eligibility, and then invited to an orientation session. After providing informed consent, they were randomized. All participants received 13 weeks of tailored weekly step goals and a Fitbit. The treatment group participants, placed in a private Twitter support group, received daily automated behavior change “tweets” informed by theory and regular automated encouragement via text to communicate with the group. Fitbit data were collected daily throughout the treatment and follow-up period. Web-based surveys and accelerometer data were collected at baseline, treatment end (13 weeks), and at 8.5 weeks after the treatment.

**Results:**

The initial study design funding was obtained from the Women’s Heart Clinic and the Stanford Clayman Institute. Funding to run this pilot study was received from the National Institutes of Health’s National Heart, Lung, and Blood Institute under Award Number K01HL136702. All procedures were approved by Stanford University’s Institutional Review Board, #32127 in 2018, prior to beginning data collection. Recruitment for this study was conducted in May 2019. Of the 858 people screened, 113 met the eligibility criteria, 68 came to an information session, and 45 consented to participate in this pilot study. One participant dropped out of the intervention, and complete follow-up data were obtained from 39 of the 45 participants (87% of the sample). Data were collected over 6 months from June to December 2019. Feasibility, acceptability, and preliminary efficacy results are being analyzed and will be reported in the winter of 2021.

**Conclusions:**

This pilot study is assessing the feasibility, acceptability, and preliminary efficacy of delivering behavior change strategies in a Twitter social support group to decrease sedentary behavior in women. These findings will inform a larger evaluation. With an accessible, tailorable, and flexible platform, Twitter-delivered interventions offer potential for many treatment variations and titrations, thereby testing the effects of different behavior change strategies, peer-group makeups, and health behaviors of interest.

**Trial Registration:**

ClinicalTrials.gov NCT02958189, https://clinicaltrials.gov/ct2/show/NCT02958189

**International Registered Report Identifier (IRRID):**

DERR1-10.2196/20926

## Introduction

### Background

Sedentary behavior is a major risk factor for heart disease and early mortality, particularly among women [1-6]. Due to technological conveniences and more office-bound occupations, prolonged sitting now accounts for over half of our waking hours [7-9]. Fortunately, a growing evidence base indicates that even small increases in light-intensity physical activity can result in cardiometabolic, physical function, and mental health benefits in women and men of any age [10-12]. Compared to physical activity interventions, relatively few interventions have addressed prolonged sitting [13,14], and there is a need for more controlled trials to specifically target reductions in objectively measured prolonged sedentary behavior [14,15], especially in women at risk or with extant heart disease [1-6].

### Social Media Interventions

Social media, in particular, or web apps that allow users to receive, generate, react to, and share content via a social network are a specific type of web-based platforms harnessed in health interventions, with modest effectiveness [16]. Different from a single bout of planned exercise, prolonged sitting occurs in multiple contexts throughout the day. Interventions with an accessible, dynamic web-based component like social media, available at any and many timepoints, may show a particular benefit for reducing sedentary behavior [17]. Not only can social media deliver in-context health information with broad reach, customizability, and easy access [18,19], it can also allow users to react and add to the content and provide social support to other users (an evidence-based behavior change technique [16,20-22]).

Twitter is a choice intervention platform, with high prevalence of use (73% of the adults in the United States use social media sites, and the majority use these sites daily [23]) and often accessed via mobile devices (80% of the Twitter users access via their mobile devices [24]). Twitter has the capability of allowing for private groups to be created that are protected from the public and even friends, making it ideal for delivering and privatizing a research intervention. Additionally, Twitter messages (called tweets) have a 280-character limit, which enables messages to be short and accessible. Often used as a supplementary aid, the potential for utilizing Twitter as a stand-alone to deliver health behavior interventions is not yet fully realized [16]. When used, engagement strongly predicts the benefits [25,26]. Tweet2Quit, a Twitter-based intervention for smoking cessation, is among the first successful interventions designed to promote smoking cessation with sustained long-term engagement and maintenance of changed behavior [27,28].

### Wearable Activity Trackers

Consumer-based wearable activity trackers provide real-time self-monitoring feedback for the consumers on their activities throughout the day. Brickwood et al [29] found a nonsignificant decrease in sedentary behavior in their meta-analysis, but in their meta-analysis, Compernolle et al [30] found interventions specifically targeting sedentary behavior and using objective self-monitoring significantly reduced sedentary time. Fitbits are used in this study, and these devices have a feature, “active hours,” which tracks the consumer’s hours of 250 steps or more (equivalent to 2 minutes of walking). A secondary benefit of using a consumer-grade device is the opportunity to track the objective physical activity of participants for the entire duration of the intervention, thereby complimenting the short-duration periods measured by accelerometers [31,32].

### Behavior Change Techniques

The CONSORT (Consolidated Standards of Reporting Trials) guidelines call for precise reporting of behavior change interventions [33]. Michie et al [34] provided a taxonomy of behavior change techniques, which provide consistency and comparability across interventions, as well as facilitate identification of successful components within an intervention. The participants in our study were divided into 2 groups: the control group that only used Fitbit and the treatment group that used Fitbit and Twitter engagement, in which selected behavior change techniques were delivered. Therefore, self-monitoring with Fitbit [30] was used for both groups of participants (see Multimedia Appendix 1 for the sample of messages, the accompanying behavior change techniques, and theoretical domains [35], the behavior change theories that informed all the messages [21,36,37], and our study categorization). To simplify characterization and comparison within our intervention, we organized the behavior change techniques we delivered via the Twitter intervention into 2 types of strategies: those that occur inside the mind, or internal strategies, and those that utilize the world outside the mind, or external strategies [38,39]. Internal strategies target one’s cognitions about the behavior to be motivated, for example, promoting a growth mindset or focusing on the anticipated benefits of the behavior. External strategies utilize the outside world to help motivate the behavior, for example, using a timer to remind oneself to move or enlisting a friend to go for a walk. We used both types of strategies in this pilot study to target moving more often or breaking up prolonged sitting.

### Tweet4Wellness Intervention

This pilot study builds upon the successful, private Twitter-based social support group intervention structure of Tweet2Quit and applies it to the less studied space of decreasing sedentary behavior, with the intervention titled as Tweet4Wellness [20]. Tweet4Wellness intervention messages are delivered daily to a private peer support group; these messages utilize behavior change techniques categorized by internal and external strategies that are shown to be effective in changing behavior and they target increased movement throughout the day [21,34,35,37,40,41]. Tweet4Wellness is paired with a wearable device (Fitbit) to facilitate objective self-monitoring. The aim of this intervention is to see if adding a social component (Tweet4Wellness) would be feasible, acceptable, and lead to greater reductions in sedentary behavior relative to self-monitoring (Fitbit) alone. A secondary benefit of using a consumer-grade device is the opportunity to track the objective physical activity of the participants for the entire duration of the intervention, thereby complimenting the short-duration periods measured by accelerometers [31,32].

### Aims of This Study

Our *primary aims* are to test the feasibility, acceptability, and preliminary efficacy of Tweet4Wellness for reducing sedentary behavior when paired with self-monitoring compared to self-monitoring alone. We hypothesized that the intervention would be feasible and acceptable for women recruited from the heart clinic. The outcomes of feasibility and acceptability were operationalized by a number of emails and phone call assistance from study staff to participants; feedback from the participants informally and via a survey on usability, likability, and suggestions for improvement; and description of the study procedure challenges. Research has yet to define the clinically relevant length of a break or length of prolonged sitting that impacts health risks, and a single measure does not adequately capture all the sedentary behavior features relevant to health [42]. Therefore, to test the preliminary efficacy on sedentary behavior outcomes, we used several measures of sedentary behavior, each capturing a different component. One is an outcome provided by Fitbit: the number of active hours or daily hours achieving over 250 steps (Fitbit’s estimate equivalent of 2 minutes of walking). We chose this measure because it is the trackable behavior each participant could self-monitor throughout the intervention. We also used the following interpretable measures proposed by Byrom et al [43] in their comprehensive coverage of sedentary behavior measurement: the maximum daily sedentary bout (longest, continuous, unbroken periods of sitting/no steps); daily weighted median sedentary bout (a measure of centrality capturing the distribution of the sedentary bouts); the total number of sedentary minutes; and the total number of steps (a measure of physical activity overall) [43]. We hypothesized that Tweet4Wellness + Fitbit group will increase their active hours (hours over 250 steps), increase their information entropy, have shorter maximum sedentary bouts and daily weighted median sedentary bouts, and fewer total number of sedentary minutes relative to baseline, compared to the Fitbit-only group.

The *secondary aims* will test the same hypotheses based on the 8.5-week follow-up period with no active intervention. The exploratory aims are within the Tweet4Wellness group. We will investigate the differences in the sedentary behavior summary measures by Twitter engagement or the number of tweets sent over the study period and by the type of behavior change strategy delivered each week (internal vs external).

## Methods

### Study Design: Design, Recruitment, and Inclusion/Exclusion Criteria

#### Trial Design

This pilot study was a 2-group randomized design. Treatment and control groups were run concurrently in time, and the study setting was largely virtual with an option for in-person orientation session attendance.

#### Recruitment

**Figure 1:**
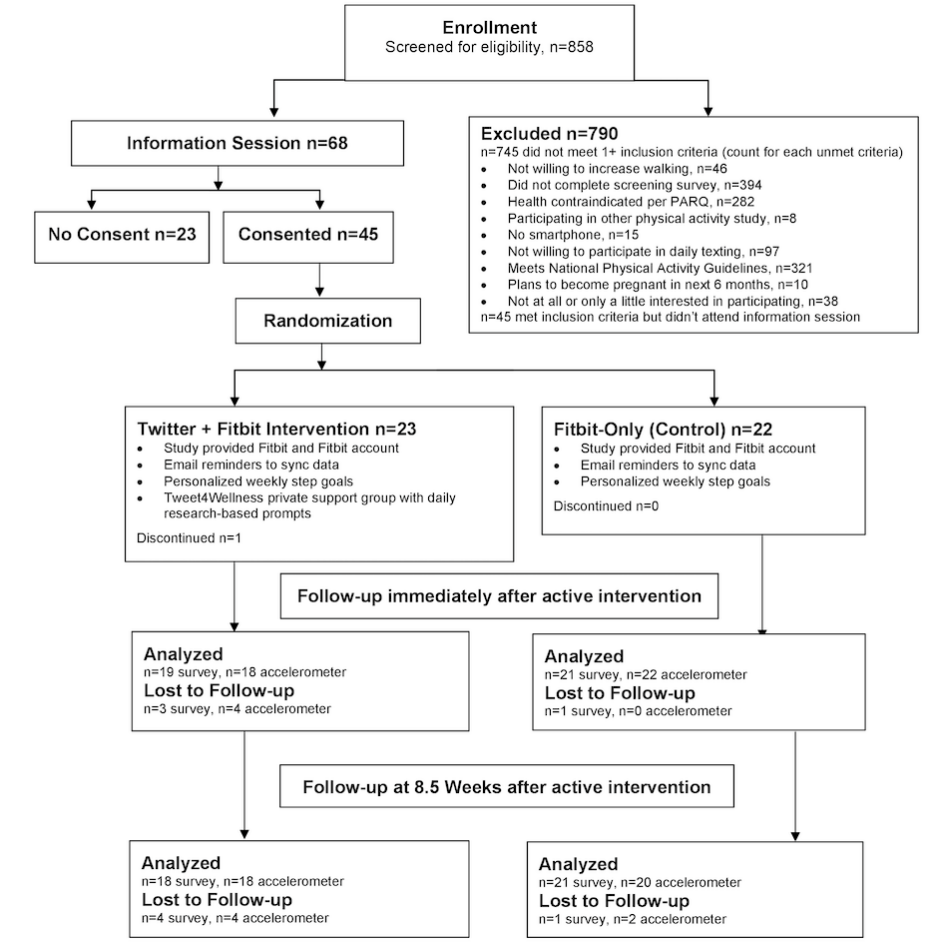
CONSORT diagram. CONSORT: Consolidated Standards of Reporting Trials.

Figure 1 shows the CONSORT diagram for this study. Participants were recruited via an email from the Stanford Research Repository System to women who had been referred to or seen at the Women’s Heart Health clinic (we did not require a diagnosis of heart disease). Recruitment emails were securely sent to women by the director of the Women’s Heart Health clinic and they were provided information about the study with a link to the screener to confirm eligibility.

#### Inclusion Criteria

The major inclusion criteria on the screener were being female, older than 18 years, willing to participate in daily tweets or texts for up to 13 weeks, having an active email account to receive study communications, having a mobile phone with unlimited texting and internet to receive text and Twitter messages, being familiar with communicating on Twitter, Facebook, or other social media (proxy for computer literacy), English-speaking (for group communication), and answer No to all 7 of the Physical Activity Readiness Questionnaire questions (physical readiness to safely perform physical activity [44]).

#### Exclusion Criteria

The major exclusion criteria were having health or physical limitations for walking (as the study encouraged walking to break up prolonged bouts of sitting) and meeting current physical activity guidelines of 150 minutes of moderate or 75 minutes of vigorous activity per week.

### Procedure

Table 1 shows the study flow from enrollment and allocation to condition (Twitter + Fitbit or Fitbit-only) through the 13-week intervention, with assessments at baseline, 13 weeks, and 21.5 weeks.

**Table 1:** Study flow.

Study activity		Study period and timeline						
		Enrollment	Allocation	Postallocation				
		0 month	0 month	1 month	2 months	3 months	4 months	5 months
**Enrollment**								
	Eligibility screen	✓						
	Information session	✓						
	Informed consent	✓						
	Randomization		✓					
**Treatments**								
	Twitter + Fitbit			✓	✓	✓		
	Fitbit only			✓	✓	✓		
**Measures**								
	Web-based surveys			✓		✓		✓
	7-day accelerometers			✓		✓		✓
	Continuous Fitbit monitoring			✓	✓	✓	✓	✓

The participants had 6 scheduled touchpoints: an orientation session (in-person or remote attendance), web-based consent and baseline survey (electronic via REDCap [Research Electronic Data Capture] [45]), a phone call with the research staff for setting up study accounts, baseline accelerometer wear (remote), posttreatment web-based survey (at 13 weeks after the baseline, electronic), accelerometer wear (mailed with a prepaid return package), and follow-up web-based survey (at 21.5 weeks after the baseline, electronic). All surveys included both closed and open-ended questions and they were emailed automatically by REDCap, with automatic reminders sent up to 2 times if participants did not respond. Participants received US $10 gift cards for completion of the 13-week posttreatment and 21.5-week follow-up surveys, and US $10 gift cards for returning the accelerometers at post and follow-up assessments. Figure 1 shows the CONSORT flow of the participants through the trial. All procedures were approved by Stanford University’s Institutional Review Board, #32127, and registered at clinicaltrials.gov, NCT02958189, prior to beginning data collection. This study was based on the most recent protocol version, June 2019. During the course of the study, there were no major study revisions.

### Orientation Session

Eligible women per the screener were contacted from the study email and invited to attend a mandatory orientation session either in-person or in a video conference. The session described the study design, timeline, and consent; explained research methods principles; and incorporated motivational interviewing techniques. The session was based on Goldberg and Kiernan’s [46] work showing increased participant retention [46,47]. One group activity had participants think through the pros and cons of being in each condition (Twitter + Fitbit or Fitbit-only) or choosing to not participate. Women who were still interested sent an email after the orientation, after which they received the electronic consent form and the baseline questionnaire. A waiver of documentation was obtained for study consent. Each page had the following sentence before moving onto the next page: “Please check here to indicate you have read and understand this information.”

For this pilot study, of the 34 women who attended the web-based orientation session, 18 (53%) consented; of the 30 women who attended the in-person session, 23 (77%) consented; and of the 4 women who had private phone call orientation sessions, all consented. The orientation session was the only in-person meeting, while the remaining parts of the study, including data collection, were remote.

### Randomization

Randomization was done in blocks with a 1:1 randomization ratio to achieve balance across the 2 conditions (Twitter + Fitbit or Fitbit-only). After 10 participants consented and completed the baseline questionnaires, they were sorted from most to least on total weekly minutes of physical activity reported on the baseline survey. The first participant was randomized to either treatment or control using a random number generator website [48], and the next participant in the pair was allotted to the other condition. The process was repeated until all of the consented participants were randomized (the last randomization in the pilot included 15 participants).

### Account Set-Up

All participants scheduled a phone call with the study staff to set up their Fitbit devices and study-provided Fitbit accounts, and they downloaded the Fitbit app on their smartphones. Participants were instructed to begin wearing the Fitbit continuously for the entire study period after the baseline accelerometer week and to open their app daily to sync their data. They could reach out to study staff via email to troubleshoot problems.

Twitter + Fitbit group participants also set up their study-provided Twitter accounts and downloaded the Twitter app on their smartphones during the call. For each Twitter account, all privacy features were turned on to prevent participants from being searched on Twitter or from having their tweets seen by people outside the treatment group. Other group norms were provided: participants were not to follow any accounts outside the private Twitter group, they were to keep their personal Twitter accounts separate from the study account, and they were instructed to use proper etiquette when messaging (no personal attacks or bullying). Tweets were monitored daily by the study staff.

### Intervention

Both the treatment (Twitter + Fitbit) and control (Fitbit-only) group received the Fitbit self-monitoring component. The treatment group also received the Twitter intervention.

#### Fitbit Self-Monitoring Component

All participants, both control (n=22) and treatment (n=23), received a study-provided Fitbit Inspire and study-provided Fitbit account connected to Fitabase, a web-based analytics and data aggregation system (Small Steps Labs). The Fitbit allowed for self-monitoring of daily steps and number of active hours. Participants were encouraged to open the Fitbit app daily to monitor their activities and to sync their data with Fitabase. When a participant did not sync their Fitbit data for over 24 hours, a study team member who monitored the Fitabase data site daily would send an email reminding the participant to open the app and sync their device. Additionally, all study participants received weekly text messages to achieve an average of 10% more steps per day, given their average step count the previous week (automatically generated and sent by the study platform). This was done to provide personalization as well as encourage data syncing (required for accurate personalized step goals). Weekly texts stopped after 13 weeks of active treatment, while Fitbit data were still collected during the follow-up period.

#### Twitter Component

Treatment participants were signed up with study-provided accounts for a private Twitter group. The study-provided account preserved participant anonymity, allowed for study control of the privacy settings within the group, allowed researchers to discontinue an account if any personal threats or harming messages were posted by a participant; and facilitated tweet captures if any direct messages between group members occurred. Twitter group participants received daily prompts suggesting a behavior change strategy and encouraging group sharing and discussion (see Multimedia Appendix 1 for examples). The behavior change strategies in the daily prompts were informed by theories of behavior change, namely, Bandura’s Social Cognitive Theory [21], Prochaska’s Transtheoretical Model of Behavior Change [49], Dweck’s Implicit Theories model [50,51], and Gollwitzer’s implementation intentions [37]. We also tied each strategy to a behavior change technique in the taxonomy for consistent language and constructs proposed by Michie et al [34]. All of the strategies were more broadly organized into 2 categories: (1) internal strategies, directed at thoughts or self-talk (eg, “by paying attention to how you feel before and after a walk, you can start to ‘show’ your brain the real-time benefits of physical activity. Each time you do this, you strengthen the connection. Try this today for your ‘move more’ walk. Share how it worked!”) and (2) external strategies, directed at changing the outside world to help achieve the behavior change (eg, “What’s your ‘slump time' of the day when you feel most rundown? Even light movement can combat it and will replace less healthful fixes (like candy!). Schedule a 5-minute walk during your slump time today and how you will remind yourself to take it. Have you tried this before?”). Having 2 categories simplified message scheduling and allowed for exploratory analyses to compare the relative effectiveness of each category (eg, did weeks with external strategies result in more sedentary behavior reduction than weeks with internal strategies?). Each week alternated between 3 sedentary behavior goals: move more (total steps per day), move more often (frequency of steps per day), and sit less (breaking up prolonged sitting). The message organization scheme is shown in Multimedia Appendix 1. Participants were encouraged to tweet in the group daily either for support or to address the prompt or both. The research staff monitored the daily activity for bullying or threatening messages.

Treatment group participants also received daily automated texts directly in their phone, providing feedback on their tweeting behavior on the prior day, praising tweeters, and encouraging nontweeters to engage. The automated text, delivered via the study web platform, considered the prior day(s)’ activity. If a participant tweeted within the previous 24 hours, they received a praise or reinforcement text at the following frequencies: every other day for weeks 1-2, every 3 days for weeks 3-6, every 4 days for weeks 7-10, and every 5 days for weeks 11-13. If a participant did not tweet within the previous 24 hours, they received an encouragement or a reminder to tweet text at the following frequencies: every day for weeks 1-4, every other day for weeks 5-10, and every 3 days for weeks 11-13. The automated text message frequency was originally scheduled for every day; however, several participants complained about the frequency and 1 participant requested that the messages stop (which was honored for that participant); therefore, a gradated schedule beginning week 5 was created for all. The frequency of encouragement texts remained higher than that of the praise texts, as encouragement has been shown to improve or increase engagement [27,28].

### Measures

#### Surveys

The survey questions assessed the participants’ goals and motivations regarding walking and sedentary behavior, access to green environments, self-efficacy to make physical activity changes, and current physical activity status (see Multimedia Appendix 2 for sample questions [21,50-55]).

#### Feasibility

Feasibility was measured in several ways. Use of the self-monitoring component, Fitbit, was measured via Fitabase, with number of days with no steps considered as nonwear/nonsynced days. Use of the Twitter intervention was determined by the number of sent tweets and number of days that the participant tweeted. The ability to recruit was measured by the proportion of the screened eligible women/women sent emails and the proportion of interested eligible women/women who attended the orientation. The acceptability of the Fitbit component was assessed for both conditions via several close-ended survey questions: some questions on support (eg, I felt I received a significant amount of support for being more active throughout the day when using Fitbit, 6-point Likert scale from strongly disagree to strongly agree) and some questions on perceived utility of the added adherence features (eg, how helpful did you find the weekly step goal texted to you, 5-point Likert scale from not at all helpful to extremely helpful). Acceptability of the Twitter component was assessed via close-ended survey questions parallel to the Fitbit questions and 2 open-ended questions: “What did you find helpful/would you change about the Twitter support group?” Emails sent and received by the study staff, troubleshooting issues, and various procedural challenges were all tracked and documented.

#### Behavioral Outcomes

Sedentary behavior was measured in 2 ways. First, participants wore triaxial accelerometers (wrist-worn Axivity AX3, [Newcastle upon Tyne, UK] or GENEActiv [Activinsights Limited, Cambridge, UK] [56]) continuously for 7 days at baseline, posttreatment, and at 8.5 weeks follow-up. Second, after the initial baseline accelerometer data collection, participants wore Fitbits continuously throughout the 21.5 weeks of the study and follow-up. The summary measures of the sedentary behavior we derive from these devices and time periods as behavioral outcomes are averaged at the day level: the number of active hours, the maximum sedentary bout length, the weighted median sedentary bout length, total sedentary minutes, and total step count.

#### Exploratory Outcomes

Engagement via tweeting was measured as the number of tweets sent and the number of days the participant tweeted. Internal versus external strategies will be separated into 2 groups, with time as a factor for analyses (week 1 internal vs week 5 internal).

### Analysis Plan for Pilot Data

We will describe the feasibility outcomes both quantitatively (descriptive statistics of survey responses and engagement data) and qualitatively (describing unexpected events). The mixed-effects models that will be used to analyze the change from baseline to postintervention and at follow-up on the sedentary behavior outcomes by condition are outlined in more detail in Multimedia Appendix 3. Exploratory aims looking within the Twitter condition only for differences by the type of strategy (internal vs external) will add strategy type as a predictor to the mixed-effects model and use the number of tweets and number of days tweeted as engagement covariates.

### Trial Sample Size/Data Safety and Privacy

#### Sample Size

Given this was a pilot, we intended to run all eligible and interested participants. The Tweet2Quit results, the Twitter-based intervention that the current Tweet4Wellness was based on, and research on active web-based participation group size [20,27,28] suggested a Twitter group size of 17-25.

#### Data Safety and Privacy

As the intervention was a low risk, a data safety monitoring board was not required. The study recorded any adverse events in the Food and Drug Administration study binder. Data were collected and kept in secure web-based databases such as REDCap [45] that are password-protected with access limited to the study team. Daily Twitter activity was monitored by the study staff.

## Results

The initial study design funding was obtained from the Women’s Heart Clinic and the Stanford Clayman Institute. Funding to run this pilot study was received from the National Institutes of Health’s National Heart, Lung, and Blood Institute under Award Number K01HL136702. All procedures were approved by Stanford University’s Institutional Review Board, #32127 in 2018, prior to beginning data collection. Recruitment for this study was conducted in May 2019. Of the 858 people screened, 113 met the eligibility criteria, 68 came to an information session, and 45 consented to participate in this pilot study. One participant dropped out of the intervention; complete follow-up data were obtained from 39 of the 45 participants (87% of the sample). Data were collected over 6 months from June to December 2019. Feasibility, acceptability, and preliminary efficacy results are being analyzed and will be reported in the winter of 2021.

## Discussion

### Principal Findings

This study intervention and design were built upon the positive findings of the Tweet2Quit smoking cessation platform [20,27,28]. In this study, we extend the intervention framework to sedentary behavior reduction in female patients at a women’s heart clinic. We investigated the additive effects of Tweet4Wellness on top of providing a Fitbit with weekly personalized step goals for reducing sedentary behavior. We tested the feasibility, acceptability, and preliminary efficacy of the Twitter + Fitbit and Fitbit-only conditions to inform a larger trial.

### Strengths and Limitations

There are several strengths to the Tweet4Wellness intervention. First, it contributes to the literature on sedentary behavior reduction, where there is a need for more randomized controlled trials that primarily focus on prolonged sitting [14]. Sedentary behavior is an independent risk factor for heart disease, particularly in women, [2,4,6] and is less studied than physical activity interventions [1,2,4,14]. Second, with a private social media group, it allows for mutual social support with the ability to automatically deliver intervention content. Third, this intervention utilizes a consumer-grade product to track daily activity throughout the entire intervention period, which provides 2 simultaneous benefits: (1) participants can self-monitor their behavior, thereby increasing their motivation to wear the Fitbit compared to accelerometers, which provide no user feedback [30,31] and (2) it provides data on the entire time course of the trial, thereby complementing the endpoints where accelerometers were used. Finally, the intervention uses behavior change theories to inform daily messages, ties messages to a commonly used taxonomy of behavior change techniques to aid in cross-study comparisons, and organizes the messages into 2 categories (internal and external) to facilitate comparison of the effectiveness within our study. Given the behavioral design, condition blinding was not feasible. Another limitation is that the conditions are not balanced for attention, as the Tweet4Wellness condition had daily intervention touchpoints. A strength of the additive design of this study is that an active treatment was offered to all who were eligible, potentially increasing enrollment and retention.

With both groups wearing Fitbits, this study has the advantage of providing rich data to fill in the gaps between standard accelerometer measurement timepoints. Challenges include the numerous issues that come with free-living data collection, and operationalizing distinctions between nonwear and sedentary behavior in the Fitbit in the absence of current research consensus. Therefore, the design purposefully has participants wearing both the triaxial accelerometer and the Fitbit during posttreatment and follow-up periods to allow for cross-comparison of device outputs.

Owing to the small sample size of this pilot study, we are not powered to test full efficacy. We instead look for trends in the sedentary behavior to serve as preliminary findings to inform a full-size powered randomized controlled trial to evaluate efficacy.

### Conclusions

If Tweet4Wellness is found to be feasible and acceptable and has some preliminary evidence of efficacy with regard to reduced sedentary behavior, these pilot findings would guide any adjustments in scaling to a full-size randomized controlled trial to evaluate efficacy. Tweet4Wellness could provide a far-reaching program for anyone to receive social support from others to reduce sedentary behavior, while also learning behavior techniques for change. Given the current shelter-in-place orders during the COVID-19 pandemic, evaluating the evidence of remote participation, health promotion platforms, and protocols is of timely value. Future studies will titrate the active ingredients in this protocol, vary the group dynamics to have mixed sex groups, and identify the optimal frequency of intervention messaging to maximize long-term engagement.

## Appendix

Multimedia Appendix 1 Behavior change techniques and theoretical domains of Twitter intervention messages.

Multimedia Appendix 2 Sample survey questions.

Multimedia Appendix 3 Description of the sedentary measures data.

## Abbreviations

CONSORT: Consolidated Standards of Reporting Trials
REDCap: Research Electronic Data Capture
