# Supplementary material for: Twitter-Based Social Support Added to Fitbit Self-Monitoring for Decreasing Sedentary Behavior: Protocol for a Randomized Controlled Pilot Trial With Female Patients From a Women’s Heart Clinic
Source: JMIR Res Protoc. 2020 Dec 4;9(12):e20926. doi: 10.2196/20926 (PMC7748950; doi:10.2196/20926)

**Multimedia Appendix 1: Behavior change techniques and theoretical domains of twitter intervention messages.**

Note: All behavior change techniques and messages we incorporated were informed by Bandura’s Social Cognitive Theory [21], Prochaska et al.’s Transtheoretical Model of Behavior Change [48], Dweck’s Implict Theories Model [49,50] and Gollwitzer’s Implementation Intentions [37].

| **Strategy Category** | **Behavior Change Technique^1^** | **Theoretical Domain: Construct^2^** | **Example Message** |
| --- | --- | --- | --- |
| Internal | - 1. Goal setting (behavior)   2. Monitoring of emotional consequences | Emotion: Positive Negative Affect; Goals: Action planning | Down moods make it hard to start physical activity, but physical activity can improve your mood and perspective. Have you experienced this? What's a small mood-movement rule you could realistically make (e.g. go for a walk when I'm down) for those times when you feel super low? |
|  | - 1. Identity associated with changed behavior   2. Self-talk | Emotion: Positive Negative Affect | Today, start paying attention to your inner dialogue around movement. What "counts" as physical activity in your mind? What feelings come to mind when you think of exercise? |
|  | - 1. Monitoring of emotional consequences | Emotion: Positive Negative Affect;  Skills: Practice | By paying attention to how you feel before and after a walk, you can start to show your brain the real-time benefits of physical activity. Each time you do this, you strengthen the connection. Try this today for your "move more" goal. Share how it worked! |
|  | 1.2 Problem solving | Behavioral regulation: Action Planning | Yesterday you shared your greatest barrier to physical activity. Today, generate at least three possible solutions to your barrier, and share them! For yourself, record the pros and cons to each possible solution. We will consider them each this week. |
|  | - 1. Mental rehearsal of past performance   2. Focus on past success | Beliefs about capabilities: Self-efficacy, Self-esteem, Beliefs | Build your movement self-esteem. Think of the last time you didn't feel like moving or being active, but somehow you were able to overcome the feeling. Share what worked for you to successfully get yourself to move. |
|  | 9.1 Credible source  15.4 Self-talk | Knowledge: Knowledge; Beliefs about capabilities: Beliefs | "All or nothing" thinking sets us up for doing nothing AND feeling bad about it. The new activity guidelines find ANY movement matters, so even if you don't have the 20 minutes for your vigorous walk, a 2-minute burst is still great! What "counts" as activity for you? |
|  | - 1. Comparative imagining of future outcomes   2. MMental rehearsal of successful performance | Beliefs about capabilities: Beliefs; Optimism: Identity | Studies show imagining your *future self* can increase your *present self*'s motivation! Take your movement minute today to imagine your ideal, active self. Consider how you would feel, gratitude for choices you made, what you'd do. Share a word or two to describe it! |
| External | - 1. Associative learning   2. Behavior substitution | Environmental context and resources: Barriers / facilitators | If you build walking into already scheduled tasks, you will be more likely to do it. What activity today you can do while walking instead of sitting (e.g. a phone call, a meeting, walking instead of driving to an errand)? |
|  | 10.2 Material reward  10.9 Self-reward | Reinforcement, Incentives: Reinforcement | Focus this week on the **effort** you spend, rather than the total time. If you spent only 5 minutes but high intensity on your walk, this "counts" even if it wasn't long. Can you think of a good reward for yourself for trying the intensity challenge this week? |
|  | - 1. Reduce prompts / cues   2. RRestructuring the physical environment | Environmental context and resources: Barriers/ facilitators; | Pictures allow for a fresh perspective. Take pictures of where you spend your most "lazy time" at home and identify things that help keep you in your seat. What changes can you make to these places to encourage more movement? |
|  | 1.1 Goal setting  1.8 Behavioral contract  7.1 Prompts/cues | Goals: Target setting, Action planning | Only have a minute? It still counts! Just a 2-minute walk to break 45 minutes of sitting can boost mental & physical health. What Movement Break goal is possible for you today? Write it down, or text it here, as your reminder. |
|  | 1.1 Goal setting  7.1 Prompts/cues | Goals: Target setting, Action planning | Share a sitting break goal for today. e.g. I will set an alarm to get up every (period of time) to get my blood flowing, maybe by stretching or maybe by taking a 2-minute walk! |
|  | 1.1 Goal setting  1.4 Action planning  7.1 Prompts/cues | Goals: Target setting, Implementation intention, Action planning | Writing down goals helps you achieve them: one study found just writing it down increased success rate by 40%! Sharing them holds you accountable! What physical activity will you do today: when will be, and where will it occur? Write it here to make it stick. |

1. Behavior change technique classified according to Michie et al.’s (2013) Behavior Change Technique Taxonomy.

2. Theoretical domain and construct classified according to Cane et al.’s (2012) Theoretical Domain Framework.

Message design scheme


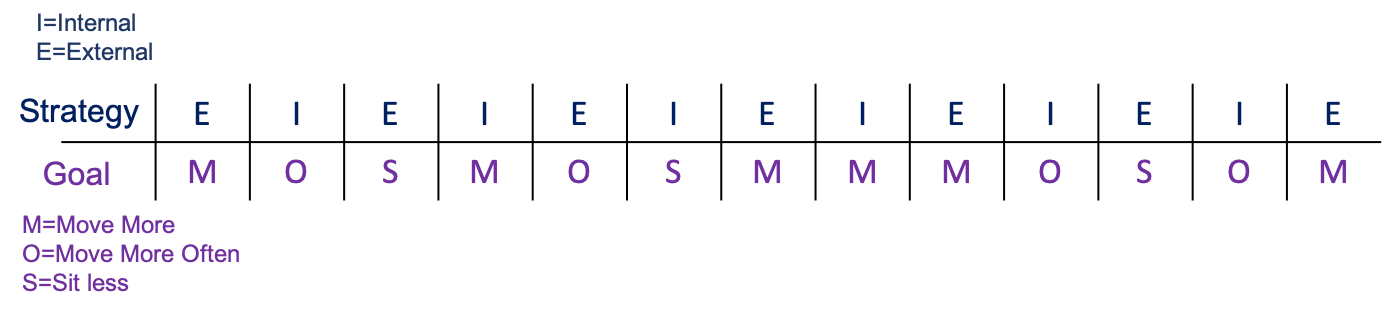

Supplement: Multimedia Appendix 1 [file resprot_v9i12e20926_app1.docx]
