# Supplementary material for: Twitter-Based Social Support Added to Fitbit Self-Monitoring for Decreasing Sedentary Behavior: Protocol for a Randomized Controlled Pilot Trial With Female Patients From a Women’s Heart Clinic
Source: JMIR Res Protoc. 2020 Dec 4;9(12):e20926. doi: 10.2196/20926 (PMC7748950; doi:10.2196/20926)
Supplement: Multimedia Appendix 2 [file resprot_v9i12e20926_app2.docx]

Sample questions based on Dweck’s Growth Mindset model and questionnaire [49].


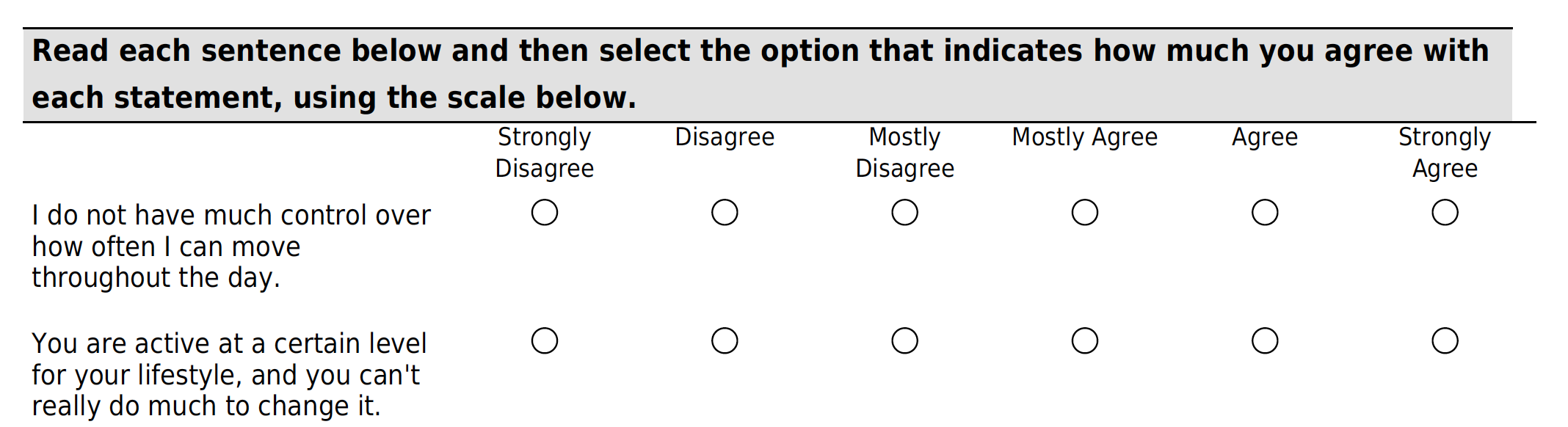


Self-efficacy question based on Bandura’s self-efficacy of exercise [21].
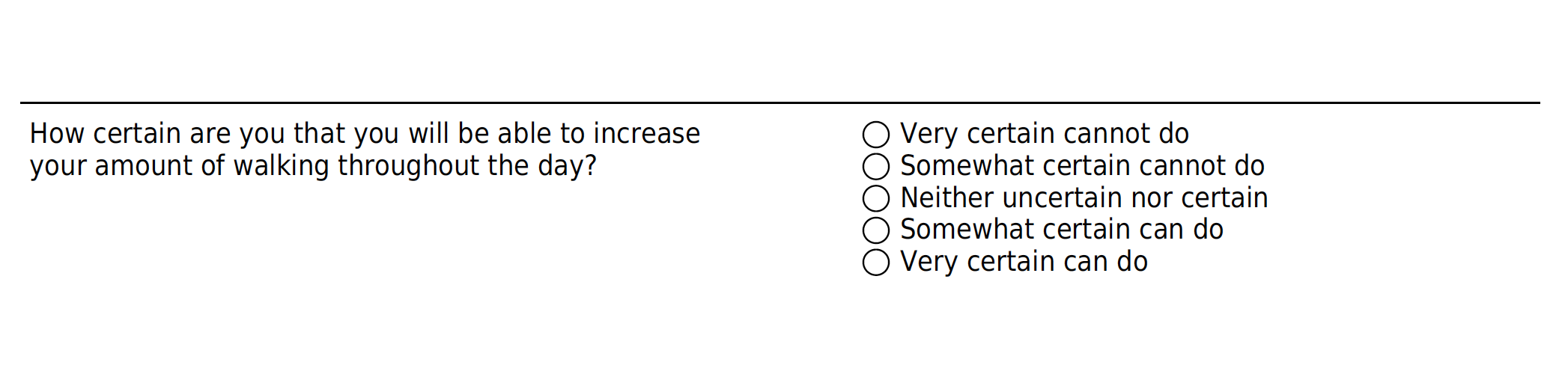


Sedentary Behavior Questionnaire, International Physical Activity Questionnaire, and questions based on Segar et al.’s exercise goal assessment were also used [51-54].
