# Supplementary material for: Twitter-Based Social Support Added to Fitbit Self-Monitoring for Decreasing Sedentary Behavior: Protocol for a Randomized Controlled Pilot Trial With Female Patients From a Women’s Heart Clinic
Source: JMIR Res Protoc. 2020 Dec 4;9(12):e20926. doi: 10.2196/20926 (PMC7748950; doi:10.2196/20926)
Supplement: Multimedia Appendix 3 [file resprot_v9i12e20926_app3.docx]

**Analysis Plan**

In our behavior analyses, we will run a mixed effects model with person as a random effect to predict each sedentary behavior outcome separately. One sedentary behavior outcome is Fitbit’s visible tracker of sedentary behavior for the user and is available on the app: number of active hours. Another, as a marker of total daily activity, is total daily steps. The others are recommended by Byrom et al., 2016. The outcomes to describe the pattern of sedentary behavior are:

1. Average daily active hours (daily hours where 250 steps or more were achieved). This is output by the Fitbit app, and 250 steps is Fitbits’ estimated equivalent of 2 minutes walking.
2. Average daily maximum sedentary bouts (maximum length of uninterrupted sitting during awake hours)
3. Average daily weighted median sedentary bout (the length of the sedentary bout corresponding to 50% of daily accumulated sedentary time. For example, if 10 h of sedentary time is recorded, this measure would represent the length of the bout that contains the 5 h time-point when bouts are ordered cumulatively from smallest to largest (see Byrom et al., 2016).
4. Average daily total sedentary minutes during awake hours (sleep determined by algorithms for the accelerometers or Fitbit’s proprietary sleep detection algorithm.
5. Average daily total steps.

Change from baseline to post intervention will be analyzed for each outcome separately, and also separated by movement tracker: accelerometer (7-days of 100 Htz triaxial accelerometer data collected at baseline, post-treatment, and follow-up during study period) and Fitbit (daily, minute-level data collected every day for entire study period). Fitbit data will also be analyzed in a time series model to explore the trajectory and pattern of change in each behavioral outcome over time.

**Table 1: Schema of statistical models for sedentary behavior outcomes**

| **Model** | **Random Effects** | **Predictors** | **Behavioral Outcome** | **Data Type** |
| --- | --- | --- | --- | --- |
| Linear mixed model regressions predicting change from baseline to post-treatment outcomes | Individual | Condition,  Age,  Engagement | Avg daily active hours | Accelerometers^1^ |
|  |  |  |  | Fitbit^2^ |
|  |  |  | Avg daily max sedentary bout | Accelerometers^1^ |
|  |  |  |  | Fitbit^2^ |
|  |  |  | Avg weighted sedentary median bout length | Accelerometers^1^ |
|  |  |  |  | Fitbit^2^ |
|  |  |  | Avg total daily sedentary minutes | Accelerometers^1^ |
|  |  |  |  | Fitbit^2^ |
|  |  |  | Avg daily total steps | Accelerometers^1^ |
|  |  |  |  | Fitbit^2^ |

^1^7 days, at baseline, post-intervention, post-follow-up, 100 Hz, triaxial data

^2^ Continuous throughout study, minute level, steps data
